# Normothermic Intraperitoneal and Systemic Treatment (NIPS) Using Paclitaxel for Peritoneal Metastases from Gastrointestinal Cancer

**DOI:** 10.3390/cancers18132166

**Published:** 2026-07-06

**Authors:** Joji Kitayama

**Affiliations:** Department of Surgical Oncology, Japan Institute for Health Security, Tokyo 162-8655, Japan; kitayama.j@jihs.go.jp; Tel.: +81-3-3202-7181

**Keywords:** peritoneal metastasis, peritoneal–plasma barrier, catheter-based intraperitoneal chemotherapy, paclitaxel, conversion surgery, NIPS

## Abstract

Peritoneal metastasis (PM) is the most frequent and lethal pattern of dissemination in gastrointestinal malignancies. Despite advances in systemic chemotherapy, outcomes remain poor because the unique biology of PM, characterized by poor vascularization and the peritoneal–plasma barrier (PPB), limits drug penetration and contributes to treatment resistance. To address these challenges, several locoregional treatment strategies have been developed, including cytoreductive surgery plus hyperthermic intraperitoneal chemotherapy (CRS + HIPEC) and pressurized intraperitoneal aerosol chemotherapy (PIPAC). However, their widespread adoption is constrained by invasiveness, strict patient selection, and inconsistent survival benefits. Normothermic intraperitoneal and systemic treatment (NIPS) has emerged as a practical and less invasive alternative, particularly in East Asia. Through an implanted intraperitoneal port, NIPS enables repeated drug administration, providing sustained regional exposure while imposing minimal procedural burden. Importantly, it can be readily integrated with systemic chemotherapy, making it suitable for long-term multimodal treatment. Among available agents, paclitaxel (PTX) is particularly well suited for intraperitoneal administration because of its prolonged retention within the peritoneal cavity and limited systemic absorption. These pharmacokinetic properties allow high local drug concentrations with relatively low systemic toxicity. Consequently, PTX-based NIPS represents a biologically rational and clinically feasible treatment strategy for PM. This review summarizes the pharmacological rationale, clinical evidence, and emerging innovations in drug formulation and delivery that may further enhance the efficacy of PTX-based intraperitoneal chemotherapy for this challenging disease.

## Simple Summary

Peritoneal metastasis (PM) is a common and highly lethal form of spread in gastrointestinal cancers. Conventional systemic chemotherapy is ineffective because poor tumor vascularization and the peritoneal–plasma barrier limit drug delivery to peritoneal tumors. Normothermic intraperitoneal and systemic treatment (NIPS) offers a practical alternative by enabling repeated drug administration through an implanted port and easy combination with systemic therapy. Paclitaxel (PTX) is particularly well suited for this approach because of its favorable pharmacokinetic properties, and accumulating clinical evidence supports the efficacy and safety of PTX-based CBIP. Future innovations may further improve outcome of the patients with PM.

## 1. Introduction

The incidence of peritoneal metastasis (PM) in gastrointestinal malignancies varies substantially according to the primary tumor origin; however, it consistently represents one of the major patterns of metastatic spread in advanced disease. In gastric cancer, synchronous PM is reported in approximately 10–21% of patients at initial diagnosis. However, when occult disease detected by peritoneal cytology, staging laparoscopy, recurrent cases, and autopsy findings are taken into account, the lifetime incidence of PM is estimated to reach 30–50% [1,2,3]. In pancreatic cancer, radiologically detectable synchronous PM is observed in approximately 9–14% of patients, whereas PM is identified in 20–50% of advanced cases when staging laparoscopy and autopsy data are included [4,5], indicating a high prevalence of occult peritoneal disease. In colorectal cancer, the incidence of synchronous PM is relatively lower, ranging from 4 to 8% at diagnosis; however, the cumulative incidence increases to approximately 8–25% [6,7]. Among hepatobiliary malignancies, gallbladder cancer and cholangiocarcinoma exhibit synchronous PM in approximately 10–20% of patients, whereas hepatocellular carcinoma shows a lower incidence of only 2–6% [8].

Despite advances in systemic chemotherapy and molecular-targeted therapy, the prognosis of patients with PM remains substantially worse than that of patients with other metastatic patterns, even within the same stage IV category [9,10,11]. This unfavorable outcome is largely attributable to the unique biological and anatomical characteristics of PM [12], including sparse vascularization [13], dense stromal fibrosis [14], the peritoneal–plasma barrier (PPB) [15], and a profoundly immunosuppressive microenvironment [16]. Specifically, the PPB critically influences drug delivery in peritoneal metastasis [15,17]. This dynamic barrier, composed of the mesothelium, interstitium, and microvasculature, enables prolonged retention of intraperitoneally administered drugs while simultaneously limiting penetration of systemically delivered agents into peritoneal tumors. Drug transport is strongly affected by molecular size, solubility, fibrosis, and extracellular matrix density [18,19]. Consequently, achieving deep drug penetration into metastatic nodules remains a major therapeutic challenge.

These anatomical and pharmacologic characteristics provide a strong rationale for intraperitoneal (IP) chemotherapy. By directly delivering anticancer agents into the peritoneal cavity, IP administration can achieve markedly higher local drug exposure while limiting systemic toxicity. Among various locoregional approaches, cytoreductive surgery (CRS) combined with hyperthermic intraperitoneal chemotherapy (HIPEC) has been widely adopted, particularly in Western countries. HIPEC combines macroscopic tumor debulking with heat-enhanced cytotoxicity and improved tissue penetration [20] and has demonstrated survival benefit in selected patients with ovarian [21] and colorectal [22] cancers. CRS+HIPEC has also been reported to be beneficial for selected patients with gastric [23,24] and pancreatic [25] cancers, although the benefit remains highly dependent on tumor biology, completeness of cytoreduction, and patient selection. Another emerging strategy is pressurized intraperitoneal aerosol chemotherapy (PIPAC), which was developed mainly in Europe. PIPAC delivers aerosolized chemotherapy under pressure to improve intraperitoneal distribution and tissue penetration, and early studies have demonstrated favorable pathological responses and promising survival outcomes [26,27,28,29]. However, the absence of randomized evidence and standardized treatment protocols remains a major limitation. Comprehensive recent reviews have summarized the current clinical experience and evidence regarding HIPEC [30,31] and PIPAC in gastrointestinal malignancies [32,33].

**Figure 1:**
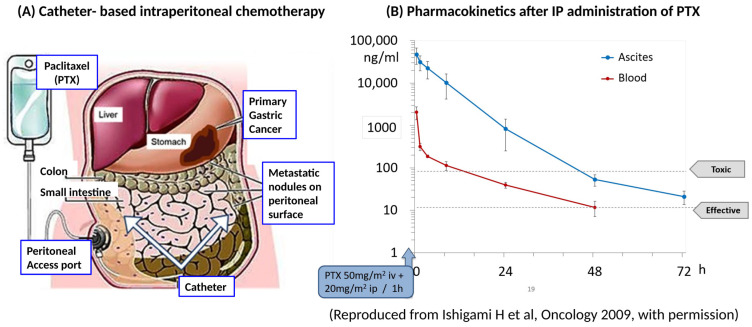
(**A**) Catheter-based intraperitoneal chemotherapy (CBIP) using an implantable port system. (**B**) Time-course changes in paclitaxel (PTX) concentrations in serum and peritoneal fluid in a representative patient following intravenous (50 mg) and intraperitoneal (20 mg) administration of PTX [34].

In contrast, repeated normothermic IP chemotherapy delivered through a subcutaneous access port and intraperitoneal catheter, referred to as catheter-based intraperitoneal chemotherapy (CBIP), represents a more practical and minimally invasive therapeutic strategy (Figure 1A). CBIP provides a platform for sustained and repeated IP drug administration via an implanted port system, enabling non-invasive weekly delivery of high local concentrations of anticancer agents over prolonged periods. Moreover, CBIP can be safely combined with various systemic therapies, which was originally coined to mean neoadjuvant intraperitoneal and systemic chemotherapy (NIPS), reflecting its initial use before CRS. However, because this strategy is also effective in patients with more advanced disease who do not undergo surgery, the acronym NIPS is used throughout this review to denote normothermic intraperitoneal and systemic treatment. In addition, the system allows serial sampling of peritoneal fluid for cytological and biomarker-based disease monitoring. Unlike HIPEC or PIPAC, NIPS does not require repeated general anesthesia and is therefore better suited for long-term treatment. In this narrative review, we reconsider the mechanistic rationale, pharmacologic basis, clinical evidence, and future perspectives of PTX-based NIPS for gastrointestinal cancers with PM. Although formal systematic review methodology or quantitative meta-analysis was not performed, this review aims to provide a comprehensive framework for the future development of PTX-based NIPS.

## 2. Characteristics of PM from a Pharmacokinetic Perspective

The peritoneal cavity is the largest enclosed compartment in the human body, lined by a mesothelial monolayer and characterized by abundant visceral adipose tissue and a unique local immune microenvironment. Despite its large surface area, effective peritoneal blood flow is remarkably low, estimated at only 60–100 mL/min (approximately 1–2% of cardiac output). This distinctive physiological feature fundamentally differentiates the peritoneum from other visceral organs and contributes to the limited efficacy of systemic chemotherapy against peritoneal malignancies [13,35].

A key determinant of drug disposition within the peritoneal cavity is the peritoneal–plasma barrier (PPB) [15,17]. Rather than a single anatomical structure, the PPB is a dynamic, multilayered interface composed of the mesothelium, submesothelial interstitium, and subperitoneal microvasculature. Drug transport across this barrier occurs through interstitial diffusion and microvascular absorption, providing the pharmacokinetic basis for intraperitoneal (IP) chemotherapy by enabling high local drug concentrations and prolonged peritoneal residence compared with intravenous administration. Classical studies identified the microvascular endothelium as the principal rate-limiting component, with transport kinetics largely determined by molecular size, charge, and solubility [17,18]. Consequently, larger molecules exhibit slower clearance and higher peritoneal-to-plasma area-under-the-curve (AUC) ratios [36,37]. In addition, the submesothelial interstitium, particularly extracellular matrix composition and collagen density, plays a critical role in regulating solute diffusion [19,38]. Recent studies have further implicated the endothelial glycocalyx, including hyaluronan, in controlling macromolecular permeability [39]. These observations highlight the PPB as a highly dynamic interface that is continuously remodeled by physiological and pathological processes. Inflammation and tumor infiltration induce angiogenesis, fibrosis, and mesothelial injury, thereby altering transport characteristics and drug distribution.

Importantly, the PPB acts bidirectionally. While it limits systemic absorption of IP-administered drugs and thereby maintains high intraperitoneal exposure, it also restricts the penetration of systemically administered agents into peritoneal tumors. This challenge is further exacerbated by tumor-associated fibrosis and desmoplasia, which increase interstitial density and pressure, creating a formidable barrier to drug diffusion from both the bloodstream and the peritoneal cavity. Therefore, the major therapeutic challenge in PM is not merely achieving high drug concentrations within the peritoneal cavity but ensuring sufficient penetration into metastatic nodules.

## 3. Pharmacological Characteristics of PTX

The pharmacokinetic rationale underlying intraperitoneal chemotherapy is to achieve sustained high locoregional drug exposure within the peritoneal cavity while minimizing systemic exposure. Although this principle applies to a variety of intraperitoneally administered agents, the extent of the pharmacokinetic advantage depends on the physicochemical properties and formulation of the individual drug. Among the available agents, PTX possesses particularly favorable pharmacokinetic properties for IP-PTX, originally isolated from the bark of Taxus brevifolia (Pacific yew), is highly hydrophobic, and is therefore formulated with Cremophor EL and ethanol. As a result, PTX forms micellar particles with a relatively large molecular diameter (approximately 10–12 nm) in solution. These micelles are absorbed predominantly through the lymphatic system, leading to prolonged retention within the peritoneal cavity and limited systemic exposure [40]. Accordingly, after IP administration, the peritoneal-to-plasma area under the concentration–time curve (AUC) ratio for PTX has been reported to be approximately 1000 [41], with more recent studies showing a range of 550–2300 [42,43], substantially higher than that observed with many hydrophilic agents. Pharmacokinetic studies have further demonstrated that intraperitoneal PTX concentrations remain above the therapeutic threshold for up to 3 days after IP administration [34,44] (Figure 1B).

In addition, PTX exerts antifibrotic effects, including inhibition of fibroblast proliferation and extracellular matrix deposition [45,46], thereby reducing postoperative peritoneal adhesion formation even after repeated IP administration. Together, these unique pharmacokinetic and biological properties distinguish PTX from many other intraperitoneal agents and enable sustained exposure of peritoneal tumor surfaces to cytotoxic drug concentrations while minimizing systemic toxicity, making PTX one of the most suitable agents for repeated intraperitoneal administration.

## 4. Intraperitoneal Route Is an Efficacious Method to Deliver PTX into Peritoneal Tumors

**Figure 2:**
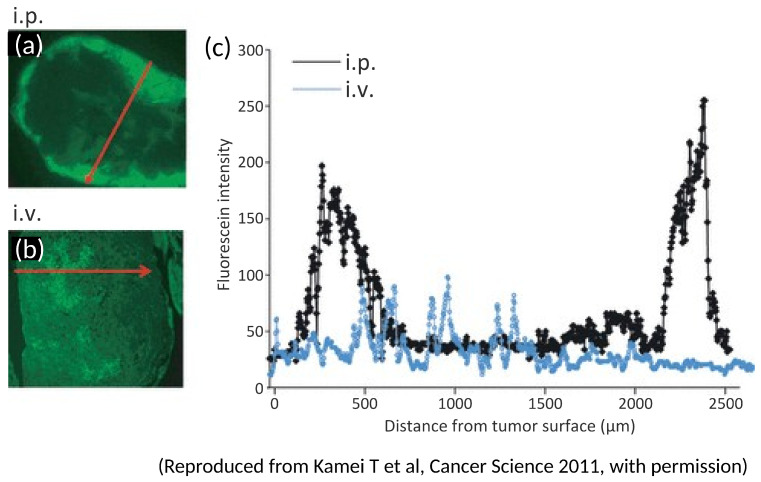
Intratumoral distribution in peritoneal tumors after intravenous (IV) or intraperitoneal (IP) injection of PTX nanoparticles. Peritoneal tumors were established by IP inoculation of the human gastric cancer cell line MKN45P. Oregon Green-conjugated PTX nanoparticles (100 μg) were administered either IV via the tail vein or directly into peritoneal cavity (IP). After 24 h, peritoneal tumors were resected, and fluorescein intensity was evaluated in tissue sections. A representative line corresponding to the deepest apparent penetration of green fluorescent PTX was defined (red arrow) (**a**,**b**), and fluorescence intensity along the line was measured and plotted (**c**). The maximal depth of PTX penetration after IP administration in each tumor is objectively measured [44].

The PPB limits drug diffusion from both the bloodstream and the peritoneal cavity; thus, the key challenge is not simply achieving high intraperitoneal drug concentrations but ensuring deep penetration into metastatic nodules. Preclinical studies have demonstrated that IP PTX achieves direct penetration into peritoneal metastatic nodules [40,41,42]. However, despite achieving high intraperitoneal concentrations, tumor penetration remains limited. Experimental studies using radiolabeled PTX have demonstrated that drug infiltration is limited to less than 100 μm from the tumor surface in xenograft models [43]. Other studies employing fluorescein-labeled PTX have shown penetration extending several hundred micrometers beneath the surface of peritoneal nodules. (Figure 2) [44]. While this superficial distribution can effectively destroy peripheral tumor cells, it is unlikely to eradicate deeply located lesions, making repeated administration essential.

**Figure 3:**
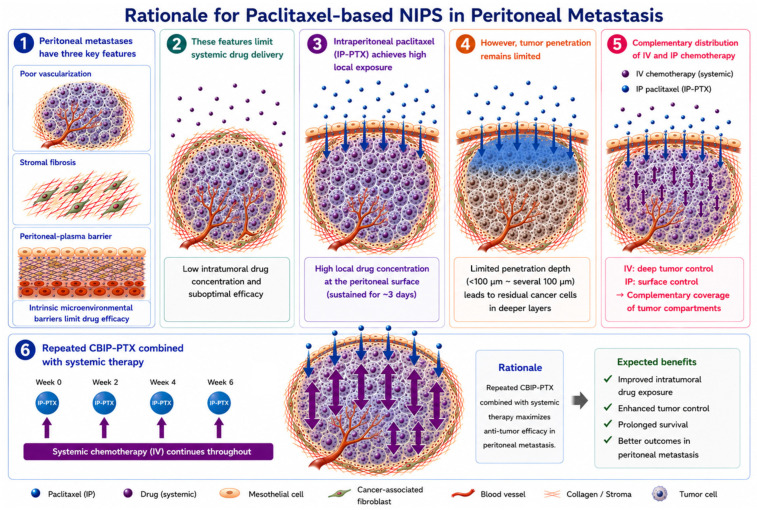
Schematic summary of the rationale for PTX-based NIPS to overcome spatial drug-delivery barriers in peritoneal metastasis.

However, even repeated IP therapy has limited penetration depth and minimal activity against extra-peritoneal disease. Despite its favorable pharmacokinetic profile, the antitumor activity of IP-PTX is constrained by its limited penetration into tumor nodules, making it most suitable for microscopic or superficial peritoneal disease rather than bulky or deeply invasive lesions. In addition, postoperative adhesions may impair drug distribution within the peritoneal cavity. These limitations support the rationale for combining IP and systemic chemotherapy as NIPS. Preclinical studies have demonstrated complementary distribution patterns, with IV-administered PTX localizing mainly to perivascular regions, whereas IP-delivered PTX accumulates in relatively avascular tumor areas [44,45] (Figure 2). In addition, PTX has been shown to induce marked destruction microvessels in the tumor periphery [46], which may disrupt tumor architecture and reduce interstitial fluid pressure, thereby facilitating deeper penetration of IV-administered agents. Recent experimental data show that IP PTX enhances the intratumoral concentration of IV administered Carboplatin [47], providing direct evidence for the synergistic potential of sequential IP and systemic chemotherapy. Collectively, these findings support NIPS using PTX as a rational strategy to overcome spatial drug-delivery barriers in PM, which is summarized in Figure 3.

## 5. Clinical Results of NIPS Using PTX for Patients with PM

### 5.1. Gastric Cancer

CBIP-PTX was developed for gastric cancer with PM (GCPM) in Japan. The first regimen consisted of oral S-1 (80 mg/m^2^, days 1–14), weekly intravenous (IV) paclitaxel (PTX, 50 mg/m^2^), and IP-PTX administered via an implanted peritoneal port. A phase I study established the recommended IP dose at 20 mg/m^2^ [34]. Early phase II studies demonstrated encouraging outcomes in patients with macroscopic PM or positive peritoneal cytology with a 1-year overall survival (OS) rate of 78% with a median survival time (MST) of 22.5 months; comparable results were reproduced in another prospective cohort [48,49]. Importantly, repeated second-look laparoscopies frequently demonstrated substantial regression of peritoneal nodules and disappearance of ascites, allowing conversion gastrectomy in selected responders. Postoperative continuation of IP chemotherapy was also feasible. Then, a randomized phase III PHOENIX-GC trial was conducted, which compared IP+IV PTX plus S-1 against standard cisplatin plus S-1 (SP) [50]. The primary analysis narrowly failed to achieve statistical significance for overall survival (median survival time [MST], 17.7 vs. 15.2 months; HR, 0.72; *p* = 0.08). Nevertheless, several exploratory and supportive analyses suggested potential benefit from IP therapy. Protocol violations complicated the interpretation of the primary analysis, including crossover to IP treatment in approximately 12% of patients assigned to the control arm. In the per-protocol population, overall survival was significantly improved with IP therapy (HR, 0.64; *p* = 0.022). In addition, the IP group included a higher proportion of patients with massive ascites, a biologically unfavorable prognostic factor, and adjustment for this baseline imbalance further strengthened the observed treatment effect (HR, 0.59; *p* = 0.008). The IP group also demonstrated superior ascites control and a higher cytological conversion rate (76% vs. 33%), supporting improved control of peritoneal disease.

Subsequent studies consistently reproduced favorable outcomes across multiple systemic chemotherapy backbones. (Table 1) Combination regimens incorporating SOX [51,52,53,54,55], FOLFOX [56,57], capecitabine/oxaliplatin or cisplatin [58,59], or S1/cisplatin [60] generally achieved 1-year OS rates of approximately 70–80% and MSTs around 15–22 months. Notably, escalation of IP-PTX doses to 40–80 mg/m^2^ did not clearly improve survival outcomes, suggesting that pharmacologic saturation within the peritoneal cavity may already be achieved at moderate doses. Most recently, the Chinese multicenter phase III DRAGON-01 trial provided further high-level evidence. In this study, IP-PTX combined with IV PTX and S-1 significantly improved survival compared with systemic chemotherapy alone, achieving an MST of 19.4 months versus 13.9 months (*p* = 0.0054). Importantly, survival benefits persisted in long-term follow-up, including improvements in 1-, 2-, 3-, and 5-year OS rates, suggesting durable disease control in selected patients [61].

One of the most clinically important aspects of IP-taxane therapy is its ability to induce cytological conversion and facilitate conversion surgery. Across studies, conversion from positive to negative peritoneal cytology (CY1 → CY0) occurred in 64–97% of patients [48,49,50,51,52,56,58,60,62], substantially exceeding rates typically achieved with systemic chemotherapy alone. Conversion gastrectomy became feasible in 26–63% of cases, and patients undergoing surgery often achieved prolonged survival, with reported MSTs ranging from 24 to 42 months [48,49,50,51,52,53,55,56,58,60,61,62]. These findings suggest that IP-PTX may convert selected stage IV patients into candidates for potentially curative-intent treatment.

The toxicity profile of CBIP-taxane appears manageable and generally comparable to conventional systemic chemotherapy. The most common adverse events are hematologic toxicities, including grade 3/4 leukopenia and neutropenia, which are usually manageable with dose modifications or temporary interruption [48,49,50,51,52,53,54,55,56,57,58,59,60,61]. Non-hematologic toxicities, such as neuropathy, gastrointestinal symptoms, fatigue, or hepatic dysfunction, are relatively infrequent. Importantly, severe abdominal pain is uncommon despite repeated IP administration. An additional issue unique to CBIP is port-related complications. In a University of Tokyo cohort, approximately 20% of patients experienced complications including inflow obstruction, infection, reflux, or fistula formation [63]. However, severe complications requiring major surgical intervention were uncommon, and most studies reported low rates of grade 3/4 port-related adverse events.

IP-PTX therapy may also have important roles beyond macroscopic peritoneal metastasis. In patients with microscopic peritoneal disease (P0CY1), several studies reported cytological conversion rates exceeding 75–95% with encouraging survival outcomes [64]. Furthermore, preliminary evidence suggests that postoperative IP-PTX may help prevent peritoneal recurrence in high-risk patients with serosal invasion [65]. Even in highly advanced cases with massive malignant ascites and poor performance status, IP therapy has shown potential benefit. Because systemic drug delivery into peritoneal lesions is particularly impaired in these patients, IP-PTX may partially overcome this limitation. Combination approaches integrating cell-free and concentrated ascites reinfusion therapy (CART) with IP-PTX have demonstrated encouraging survival outcomes despite historically dismal prognoses [66].

In Western populations, clinical evidence remains limited but is steadily emerging. In the United States, the prospective phase II STOPGAP study [67], evaluating IP-PTX in combination with 5-fluorouracil and leucovorin, has been completed and has subsequently progressed to a phase III trial (STOPGAP II) (https://clinicaltrials.gov/study/NCT07001748, accessed on 2 July 2026). In Europe, a phase III study, IPa-Gastric trial, is currently evaluating the added benefit of IP-PTX to standard fluoropyrimidine- and oxaliplatin-based systemic regimens (https://clinicaltrials.gov/study/NCT07304271, accessed on 2 July 2026). These studies are expected to clarify the generalizability of IP taxane-based strategies beyond East Asian populations and to better define their role within global treatment paradigms.

### 5.2. Pancreatic Cancer

Pancreatic ductal adenocarcinoma (PDAC) remains one of the deadliest malignancies, with a 5-year survival rate below 10% [68]. Peritoneal metastasis (PM) is a frequent and highly lethal metastatic pattern, with reported median survival of only 2–3 months and 1-year overall survival (OS) rates of 10–14% [69]. Although modern systemic regimens such as FOLFIRINOX and gemcitabine plus nab-paclitaxel (GEM+nab-PTX) have improved outcomes in metastatic PDAC [70], their efficacy specifically against PM remains unclear. In contrast, HIPEC has been poorly investigated in PDAC because of the aggressive biology and poor prognosis of the disease.

As shown in Table 2, two phase II studies have suggested promising activity of CBIP using PTX. Satoi et al. evaluated IV/IP PTX plus S-1 and reported a median OS of 16.3 months, with response and disease control rates of 36% and 82%, respectively [71]. Patients who underwent conversion surgery achieved significantly longer survival than those who did not (27.8 vs. 14.2 months). Yamada et al. subsequently investigated IV gemcitabine and nab-PTX combined with IP PTX, demonstrating a median OS of 14.5 months, a response rate of 50%, and a disease control rate of 95%, with cytology conversion observed in 39% of patients [72]. Conversion surgery was again associated with markedly prolonged survival.

Based on these encouraging findings, an ongoing phase III trial is comparing IP PTX–based therapy with standard systemic GEM+nab-PTX in patients with PDAC and PM [74]. This multicenter study will directly evaluate whether bidirectional chemotherapy using IP PTX can improve survival outcomes in this highly refractory disease.

### 5.3. Colorectal Cancer

Colorectal cancer (CRC) accounts for approximately 10.2% of all newly diagnosed cancers worldwide and is the second leading cause of cancer-related death [75]. PM is one of the most frequent metastatic patterns in CRC and is associated with particularly poor prognosis. Population-based studies have shown that synchronous PM is present in approximately 4–7% of patients at the time of initial CRC diagnosis, while an additional 4–6% develop metachronous PM during the disease course [76,77]. Historically, PM from CRC has been regarded as a terminal condition with substantially worse prognosis than hematogenous metastases such as liver or lung metastases. Large registry analyses reported median OS of only 5–9 months in untreated patients [78] and approximately 12–16 months with modern systemic chemotherapy alone [79].

In selected patients, however, CRS plus HIPEC has significantly improved outcomes. The randomized trial by Verwaal et al. demonstrated superior survival with CRS+HIPEC compared with systemic chemotherapy alone [80], and subsequent cohort studies reported median OS exceeding 30–40 months with 5-year survival rates of 30–45% after complete cytoreduction [81,82]. Prognosis is strongly influenced by PCI, completeness of cytoreduction, tumor biology, and histological subtype. Patients with low PCI and complete cytoreduction achieve the best outcomes, whereas signet-ring cell carcinoma and extensive small bowel involvement are associated with poor survival [83]. Nevertheless, recurrence remains frequent, and many patients are not candidates for aggressive surgery because of diffuse disease or poor performance status.

PIPAC has emerged as a promising alternative for CRC with PM. Recent studies have shown that oxaliplatin-based PIPAC is feasible, safe, and well tolerated. Lurvink et al. reported median OS of 15–27 months [84], while Sleiman et al., in an analysis of 949 patients from 11 studies, reported median OS ranging from 8 to 37.8 months, with most patients completing the planned treatment cycles [85]. These findings are consistent with the pooled median OS of approximately 16 months reported by Alyami et al. [26]. Histopathologic response rates were also encouraging, including complete response in nearly 50% of patients and major regression in 29% [84]. Toxicity was generally mild, with most adverse events limited to grade 1–2 abdominal pain or nausea, whereas grade ≥ 3 toxicities occurred in only 12–15% of patients and no treatment-related deaths were reported [26,85]. Quality of life was largely preserved during treatment.

NIPS using PTX has been employed in Japan for treatment of PM from CRC. A preceding phase I study demonstrated favorable safety and preliminary efficacy, with a response rate of 25%, improvement in PCI in 50% of patients, complete conversion to negative peritoneal cytology, median progression-free survival (PFS) of 8.8 months, and median OS of 29.3 months (Table 2) [73]. Based on these encouraging results, the subsequent iPac-02 study was conducted as a single-arm, multicenter phase II trial enrolling 38 patients with isolated unresectable colorectal PMs [86]. Patients received weekly intraperitoneal PTX (20 mg/m^2^) combined with FOLFOX- or CAPOX-bevacizumab. The primary endpoint was response rate, while secondary endpoints included PFS, OS, PCI improvement, conversion to negative peritoneal cytology, and safety.

## 6. Future Improvement of NIPS Using PTX for PM

### 6.1. Seek the Optimal Systemic Chemotherapy to Combine with IP-PTX

IP-PTX should be recognized fundamentally as a locoregional treatment, with limited activity against extraperitoneal disease. Given that PM frequently coexists with systemic dissemination, the integration of effective and well-tolerated systemic chemotherapy is essential to optimize overall patient outcomes. In this context, combination strategies incorporating contemporary molecularly targeted agents and immunotherapies are particularly promising. In this context, recent phase II studies have incorporated IP-PTX into modern systemic regimens, thereby enabling the concurrent use of biomarker-driven therapies, including anti-PD-1 inhibitors and HER2-targeted agents. Kang et al. reported outcomes in 22 patients with GCPM treated with FOLFOX with or without nivolumab in combination with IP-PTX (60 mg/m^2^), demonstrating a median overall survival of 20.2 months [87]. Yang et al. are currently investigating a combination regimen comprising cadonilimab (a dual CTLA-4/PD-1 inhibitor), LM-302 (a claudin 18.2-targeted antibody–drug conjugate), and S-1 with IP-PTX (20 mg/m^2^) in patients with CLDN18.2-positive GC (NCT06519591). Claudin 18.2 has emerged as a promising molecular target in gastric cancer, and its monoclonal antibody, zolbetuximab, has recently been approved as a first-line treatment for metastatic disease [88,89]. Claudin 18.2 is preferentially expressed in diffuse-type and poorly differentiated gastric cancers, histological subtypes that are strongly associated with peritoneal dissemination [90,91]. Therefore, combining IP-PTX with zolbetuximab represents a biologically rational therapeutic strategy and may provide excellent efficacy in patients with GCPM. In addition, a phase II trial (NCT05185947) is evaluating the combination of IP/IV PTX with the tyrosine kinase inhibitor nilotinib across multiple malignancies, including, but not limited to, gastric cancer [92]. These ongoing efforts are expected to refine the optimal systemic partners for IP-PTX-based therapy and to further enhance clinical outcomes through a rational, multimodal treatment approach.

### 6.2. Modification of PTX for IP-Specific Drug

Another promising strategy for improving IP chemotherapy is the development of IP-specific anticancer formulations. Increasing preclinical evidence suggests that advanced biomaterials, including hydrogels, nanoparticles, and implantable delivery systems, can achieve sustained locoregional drug release within the peritoneal cavity, thereby enhancing therapeutic exposure while reducing systemic toxicity [93]. For paclitaxel (PTX), both chemical modification and formulation-based approaches have demonstrated superior antitumor efficacy compared with conventional formulations in murine PM models. Li et al. showed that PTX solubilized with poly(L-glutamic acid) improved biodistribution and drug delivery efficiency in ovarian cancer PM [94]. Yamada et al. reported that hyaluronic acid (HA)-based formulations prolonged intraperitoneal retention of PTX and enhanced antitumor effects in nude mice [95]. Similarly, Bajaj et al. demonstrated that HA-based in situ crosslinkable hydrogels improved PTX retention and therapeutic efficacy after IP administration [96]. Soma et al. developed a nanomicellar PTX formulation using a PMB polymer, an amphiphilic copolymer composed of 2-MPC and n-BMA, which enhanced direct penetration into peritoneal tumors [45]. Emoto et al. further showed that NK105, a “core–shell”-type polymeric micellar PTX nanoparticle, enhanced antitumor activity against both peritoneal and extraperitoneal lesions [97]. In addition, De Clercq et al. demonstrated that PTX-loaded genipin-crosslinked gelatin microspheres prolonged intraperitoneal drug retention and improved therapeutic outcomes in advanced ovarian cancer models. More sophisticated nanocarrier systems, including pH-sensitive polymersomes and tumor-penetrating peptide (iRGD)-functionalized nanoparticles, have further improved tumor accumulation and intratumoral penetration after IP administration [98,99]. Collectively, these studies suggest that physicochemical modification of nanomicellar structures, including particle size, surface charge, and solvent polymer characteristics, critically influences the therapeutic efficacy of IP-PTX. Although no anticancer agents have been specifically approved yet for IP chemotherapy, the development of optimized nanodrugs for locoregional delivery may represent a major breakthrough in the treatment of PM.

### 6.3. Modification of Intraperitoneal Immune Microenvironment

Another important consideration in NIPS is its impact on the peritoneal immune microenvironment. The high local concentrations achieved by IP-PTX may exert substantial cytotoxic effects not only on tumor cells but also on resident and infiltrating immune cells, potentially resulting in profound local immunosuppression. The peritoneal cavity harbors a unique immune milieu that is generally immunosuppressive and plays a critical role in regulating tumor progression and therapeutic response in PM [16,100,101].

Recent analyses of ascitic immune cells have shown that IP-PTX treatment reduces the proportion of CD8(+) T cells while increasing CD11b(+) myeloid cells. Notably, a marked expansion of CD16(−)CCR3(+) eosinophils was observed in a subset of patients and was associated with enhanced tumor regression and improved survival outcomes [102]. These findings suggest that the therapeutic effects of IP-PTX may be mediated, at least in part, through modulation of the local immune environment. Given the pivotal role of the immune microenvironment in determining chemosensitivity and treatment outcomes [103], strategies aimed at restoring or enhancing antitumor immunity within the peritoneal cavity may represent an important avenue for maximizing the efficacy of NIPS.

Future advances are also expected to arise from a better understanding of the peritoneal microenvironment, including tumor-associated fibroblasts, stromal remodeling, molecular heterogeneity, desmoplastic stromal remodeling, and molecular heterogeneity identified by recent proteomic analyses [104,105]. These biological insights may facilitate patient stratification and the development of novel combination strategies to enhance the efficacy of regional therapies, including PTX-based NIPS.

## 7. Conclusions

NIPS using PTX represents a biologically rational treatment strategy for PM from gastrointestinal cancers, and accumulating clinical evidence increasingly supports its therapeutic utility, particularly in East Asian populations. However, much of the available evidence is derived from single-arm or non-randomized studies that are inherently susceptible to selection bias and lack appropriate comparator arms. Moreover, the current level of evidence differs substantially among gastrointestinal malignancies. Gastric cancer has the strongest clinical evidence, supported by two randomized phase III trials and multiple prospective studies. In pancreatic cancer, preliminary phase II studies have demonstrated encouraging feasibility and efficacy, although confirmatory randomized trials are still lacking. For colorectal cancer, the available evidence remains exploratory, and CRS/HIPEC and PIPAC currently represent the more established regional treatment approaches in appropriately selected patients. Therefore, the clinical role of NIPS should be carefully interpreted according to the strength of evidence available for each tumor type rather than generalized across all gastrointestinal malignancies.

Table 3 summarizes the characteristics of NIPS in comparison with HIPEC and PIPAC. A major advantage of this approach is its repeatability, which enables prolonged locoregional treatment of peritoneal metastases. Moreover, it can be safely combined with various systemic therapies, including recently developed molecularly targeted agents and immunotherapies. Although further optimization of treatment protocols and high-quality clinical evidence are still needed, PTX-based NIPS has the potential to become an integral component of future multimodal treatment strategies for gastrointestinal cancers with PM.

## Figures and Tables

**Table 1:** Efficacy of NIPS using PTX for patients with PM from gastric cancer.

Author, Year	PTX Dose (mg/m^2^)	Systemic Regimen	Study	*n*	MST	1y-OS	Response Rate	Cytology Conversion Rate	Conversion Surgery Rate	Grade 3/4 Neutropenia	Major Grade 3/4 Non-Hematological Toxicity	Ref.
Ishigami H, 2010	20	S-1+PTX	P2	40	22.5M	78.0%	10/18 (55.6%)	86.0%	37.5%	38.0%	Nausea vomit: 8%, Anorexia: 5%	[48]
Yamaguchi H, 2013	20	S-1+PTX	P2	35	17.6M	77.1%	5/7 (71.4%)	97.0%	31.4%	34.0%	Nausea vomit: 3%	[49]
Ishigami H, 2018	20	S-1+PTX	P3	114	17.7M	71.9%	9/17 (52.9%)	95.0%	45.7%	50.0%	Nausea vomit: 10%, Anorexia 10%, Febrile neutropenia 8%	[50]
Fujiwara, 2016	40	S-1+L-OHP	P2	60	ND	71.5%	4/6 (66.7%)	71.0%	35.9%	50.0%	Anorexia 12%, Febrile neutropenia 6%	[51]
Saito S, 2021	40	S-1+L-OHP	P2	44	25.8M	79.5%	2/3 (66.7%)	86.0%	45.0%	39.0%	Febrile neutropenia 5%	[52]
Chia, DKA, 2023	40	Cap+L-OHP	P2	44	14.6M	67.8%	ND	70.5%	29.5%	18.0%	Febrile neutropenia 14%	[58]
Tu, L, 2023	80	S-1+L-OHP	P2	49	16.9M	81.6%	21/47 (51.2%)	ND	26.5%	40.8%	Nausea vomit: 22.5%, Diarrhea 12.2%	[55]
Yang ZY, 2022	20	S-1+PTX	P2	67	19.3M	67.2%	22/64 (34.4%)	67.2%	62.9%	26.9%	Liver dysfunction 9%	[62]
Zhao S, 2022	80	mFOLFOX	P2	29	11.0M	ND	11/29 (37.9%)	ND	ND	34.5%	Diarrhea 20.7%	[57]
Seo WL, 2025	80	Cap+CDDP	P2	28	ND	76.9%	10/24 (41.7%)	ND	ND	41.7%	ND	[54]
Kobayashi D, 2024	20	S1+CDDP	P2	53	19.4M	73.6%	ND	64.0%	30.0%	24.0%	Diarrhea 13%, Anorexia 17%	[60]
Yan C, 2026	20	S-1+PTX	P3	148	17.7M	69.6%	ND	ND	50.7%	19.9%	Fatigue 7.7%, Anorexia 5.1%	[61]

Abbreviations: PTX: Paclitaxel, L-OHP: oxaliplatin, CDDP: cisplatin, Cap: Capecitabine, ND: Not described.

**Table 2:** Efficacy of NIPS using PTX for patients with PM from pancreatic or colorectal cancers.

Author, Year	PTX Dose (mg/m^2^)	Systemic Regimen	Study	*n*	MST	1y-OS	Response Rate	Cytology Conversion Rate	Conversion Surgery Rate	Grade 3/4 Neutropenia	Major Grade 3/4 Non-Hematological Toxicity	Ref.
Pancreatic cancer												
Satoi S, 2017	20	S-1+PTX	P2	33	16.3M	62.0%	12/33 (36.4%)	55.0%	24.2%	42.0%	Febrile neutropenia 14% Anorexia 12%	[71]
Yamada S, 2020	20	GEM+nab-PTX	P1/2	46	14.5M	61.0%	21/46 (45.6%)	76.0%	39.1%	69.6%	Anorexia 20%	[72]
Colorectal cancer												
Murono K, 2019	20	mFOLFOX6bmab or CapeOX+bmab	P1/2	6	29.3M	ND	3/6 (25%)	ND	ND	16.7%	Nausea vomit: 16.7%, Abdominal pain: 16.7%	[73]

Abbreviations: GEM: Gemcitabin, nab-PTX: Nanoparticle albumin-bound paclitaxel, Bmab: Bevasizuimab, ND: Not described.

**Table 3:** Comparative Features of HIPEC, PIPAC, and NIPS for the Management of PM.

Characteristic	HIPEC	PIPAC	NIPS
Treatment setting	During cytoreductive surgery (CRS)	Repeated laparoscopic procedure	Repeated catheter-based intraperitoneal chemotherapy
Treatment objective	Eradication of residual microscopic disease after CRS	Disease control in unresectable peritoneal metastases	Long-term regional chemotherapy combined with systemic therapy
Invasiveness	High	Moderate	Low
General anesthesia	Required	Required	not required
Repeatability	Limited	Moderate (every 6–8 weeks)	Excellent (weekly or biweekly)
Drug distribution	Heated liquid perfusion during surgery	Pressurized aerosol	Liquid intraperitoneal infusion
Tumor penetration	Moderate (heat-enhanced)	Moderate (pressure-enhanced)	Limited; mainly superficial (<100–1000 μm)

## Data Availability

No new data were created.
